# Type 1 Diabetes and Cardiovascular Risk: The Current Management Strategies

**DOI:** 10.31083/RCM46826

**Published:** 2026-05-26

**Authors:** Rohit Jacob Manoj, Aarthy Aravind, Cornelius J Fernandez, Joseph M Pappachan

**Affiliations:** ^1^Department of Internal Medicine, Aster DM Healthcare, 8703 Dubai, U.A.E.; ^2^Department of Medicine, University of Buckingham Medical School, MK18 1EG Buckingham, UK; ^3^Department of Endocrinology and Metabolism, Pilgrim Hospital, United Lincolnshire Hospitals NHS Trust, PE21 9QS Boston, UK; ^4^Department of Endocrinology and Metabolism, Countess of Chester Hospital NHS Trust, CH2 1UL Chester, UK; ^5^Faculty of Science, Manchester Metropolitan University, M15 6BH Manchester, UK; ^6^Department of Endocrinology, Kasturba Medical College, Manipal, Manipal Academy of Higher Education, 576104 Manipal, India

**Keywords:** type 1 diabetes mellitus, cardiovascular disease, metabolic control, cardiovascular disease risk, double diabetes

## Abstract

Cardiovascular disease (CVD) is the most common cause of morbidity and excess mortality in patients with diabetes. However, CVD risk varies across the different forms of diabetes mellitus owing to underlying pathobiological mechanisms. In the asymptomatic phase of prediabetes and early stages of type 2 diabetes mellitus (T2DM)—the most common form of diabetes—CVD may already be established; meanwhile, the first diagnosis of T2DM in some patients may be established only when the patients are evaluated for risk factors contributing to CVD. In contrast, type 1 diabetes mellitus (T1DM) typically presents with symptoms at the disease onset and is usually associated with a low prevalence and risk for CVD at the time of diagnosis. With good metabolic control, the CVD risk in patients with T1DM can be mitigated to some extent. While the pathophysiology and prognosis of CVD among patients with T2DM have been extensively studied and are well characterized, important uncertainties remain regarding these aspects in patients with T1DM. This clinical update review compiles the current evidence base for the evaluation and management of CVD in patients with T1DM.

## 1. Introduction

Cardiovascular disease (CVD) is a leading cause of morbidity and mortality in type 1 diabetes mellitus (T1DM), impacting both life expectancy and quality of life [1]. Although T1DM accounts for only a minority of the global burden of diabetes mellitus, its nearly universal early onset and lifelong course result in prolonged exposure to hyperglycaemia and related metabolic disturbances, which are central drivers of vascular complications [2]. Long-term glycaemic control is the strongest predictor of CVD outcomes, with trials demonstrating a lasting “legacy effect” of lower glycosylated haemoglobin (HbA1c) [3]. Nevertheless, severe hypoglycaemia is now considered a preventable risk factor for cardiovascular morbidity and mortality [4, 5]. Notably, women with T1DM lose the usual cardiovascular protection observed in the general female population and often experience greater excess risk than men, influenced by both sex-specific biological and psychosocial factors [6, 7]. Beyond hyperglycaemia, residual risk is amplified by hypertension, dyslipidaemia, insulin resistance, obesity, and renal dysfunction [8]. Novel prognostic markers and tools are increasingly recognised. Glucose metrics, such as time-in-range and variability, provide incremental value. At the same time, elevated remnant cholesterol and the presence of metabolic dysfunction-associated steatotic liver disease (MASLD) contribute substantially to the atherosclerotic burden [9, 10, 11]. Imaging techniques, including coronary artery calcium scoring and computed tomography (CT) angiography, enable earlier detection of subclinical atherosclerosis and refinement of risk assessment [12, 13]. Importantly, adults with late-onset T1DM also face increased CVD and mortality risk, suggesting the need for early and sustained preventive strategies across the lifespan [14]. Recent guidelines like the American Diabetes Association (ADA) Standards of Care 2025 emphasise comprehensive management encompassing lipids, blood pressure, kidney health, smoking cessation, and psychosocial support to mitigate this disproportionate cardiovascular burden [15].

## 2. Pathophysiology of CVD in Type 1 Diabetes Mellitus

The pathophysiology of CVD in T1DM is multifactorial and distinct from type 2 diabetes mellitus (T2DM). The main defect in T1DM is autoimmune-mediated depletion of pancreatic beta cells, leading to insulin deficiency and chronic hyperglycaemia from an early age [2]. Persistent hyperglycaemia induces endothelial injury through oxidative stress, inflammation, nitric oxide depletion, activation of advanced glycation end products (AGEs) and their receptors (RAGE), and endothelial-to-mesenchymal transition, all of which accelerate atherosclerosis [16, 17]. Glycaemic variability and recurrent hypoglycaemia exacerbate this risk by triggering pro-inflammatory, pro-thrombotic, and arrhythmogenic responses [18].

Over time, many individuals with T1DM also develop insulin resistance, often termed “double diabetes”, which is associated with obesity, hypertension, dyslipidaemia, and low-grade inflammation, further amplifying vascular injury [19, 20]. Lipoprotein biology in T1DM is unique: high-density lipoprotein (HDL) particles demonstrate impaired function due to glycation and oxidation; apolipoprotein B (*ApoB*)-containing particles and remnant cholesterol contribute disproportionately to risk; and imbalances between *ApoB-containing lipoproteins* and large HDL subspecies have been linked to atherosclerosis [21, 22].

Vascular repair mechanisms are impaired in T1DM. Molecular-level changes, including endothelial glycocalyx destruction and depletion of endothelial progenitor cells, impact vascular recovery, leading to both microvascular and macrovascular complications [23, 24]. Albuminuria reflects systemic endothelial dysfunction, and reducing albuminuria is associated with lower cardiovascular risk [25, 26].

Diabetic cardiomyopathy, driven by fibrosis, microvascular dysfunction, and chronic inflammation, predisposes to heart failure (HF) in T1DM [27]. Cardiac autoimmunity, including the presence of cardiac-specific autoantibodies, predicts later CVD events and subclinical myocardial dysfunction, highlighting immune-mediated myocardial injury as a unique pathway in T1DM [28, 29]. Autonomic neuropathy and microvascular complications (retinopathy, nephropathy) further amplify CVD risk and prognosis [30, 31].

Taken together, the cardiovascular pathology of T1DM is distinguished from that of T2DM by its earlier onset, the primacy of chronic hyperglycaemia in the pathogenesis of CVD, and the contribution of autoimmune and immune-inflammatory mechanisms, reinforcing the need for tailored preventive and therapeutic strategies.

**Fig. 1.**
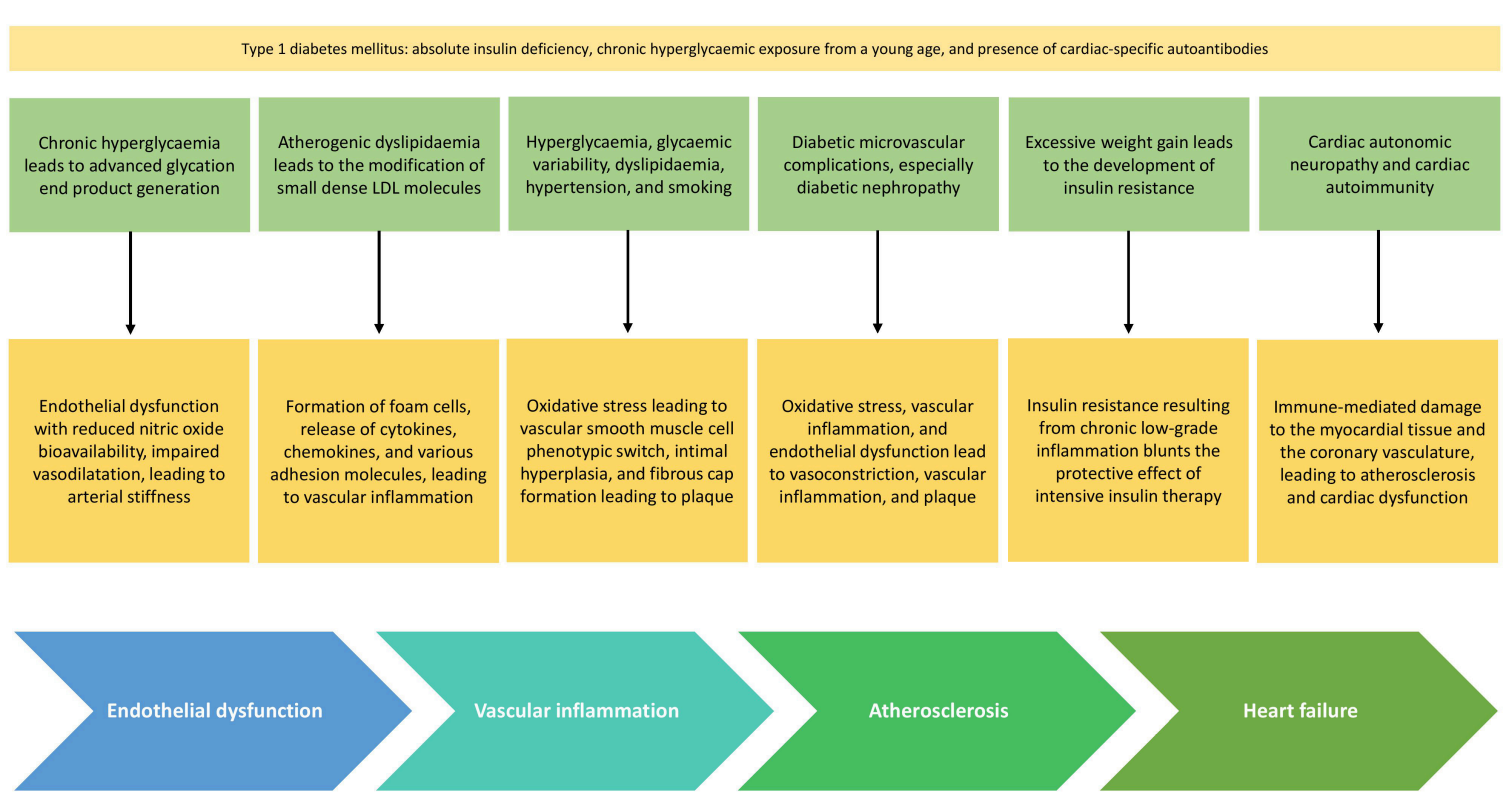
**Mechanisms for CVD among patients with T1DM**. CVD, cardiovascular disease; T1DM, type 1 diabetes mellitus; LDL, low-density lipoprotein.

Putative mechanisms for CVD among patients with T1DM are depicted in Fig. 1.

## 3. Spectrum of Cardiovascular Outcomes in Type 1 Versus Type 2 Diabetes Mellitus

T1DM and T2DM are associated with the full spectrum of atherosclerotic cardiovascular disease (ASCVD) and HF, but the patterns of risk and timing differ substantially. Individuals with T1DM typically experience earlier onset and higher relative risk of cardiovascular events compared to the general population [32].

In T1DM, myocardial infarction (MI) and stroke often occur at younger ages, reflecting the cumulative effects of long-term hyperglycaemia and early onset of risk factors. The excess burden is particularly pronounced in women and in those diagnosed during childhood or young adulthood [6, 14]. HF represents a primary cardiovascular outcome in T1DM, with risk not fully explained by traditional atherosclerotic pathways. Reviews and meta-analyses confirm that both HF with reduced and preserved ejection fraction are more prevalent in T1DM than in the general population [27, 33].

Peripheral arterial disease (PAD) and lower extremity arterial disease are also clinically significant in T1DM. Their risk correlates closely with HbA1c, blood pressure, lipids, and smoking, though contemporary data suggest that T1DM may confer comparatively lower risk of aortic aneurysm than non-diabetic populations [34]. Additionally, atrial fibrillation (AF) risk is elevated in T1DM compared to non-diabetic peers [35, 36]. Adult-onset T1DM is increasingly recognised as a group at significant risk of major adverse cardiovascular events (MACE) and mortality [37].

Collectively, while both T1DM and T2DM confer an excess cardiovascular risk, T1DM is characterised by an earlier onset, a higher relative risk, and distinct contributors, such as autoimmune and non-atherosclerotic mechanisms.

## 4. Risk Factors in the Aetiopathogenesis of Cardiovascular Disease in T1DM

### 4.1 Hyperglycaemia

Chronic hyperglycaemia is the central driver of CVD risk in T1DM. Persistent elevations in blood glucose induce oxidative stress, build-up of AGEs, systemic inflammatory processes, and endothelial dysfunction, accelerate atherosclerosis and microvascular injury [38, 39]. The DCCT/EDIC trial suggested that early, intensive glycaemic control reduces long-term cardiovascular events, establishing the concept of the “legacy effect” of HbA1c [5]. Importantly, even with optimal control, residual cardiovascular risk remains, highlighting the contribution of non-glycaemic pathways [39, 40].

### 4.2 Hypoglycaemia and Glucose Variability

Severe hypoglycaemia is now recognised as a cardiovascular risk factor in T1DM, associated with arrhythmias, myocardial ischemia, and mortality. Glycaemic variability further exacerbates vascular injury by triggering oxidative stress, inflammation, and pro-thrombotic responses. These dynamic glucose fluctuations help explain why patients with similar HbA1c values may have differing CVD risks [40]. Within T1DM, a particularly vulnerable subgroup is those with “brittle” or labile diabetes, characterised by extreme glycaemic lability with frequent, unpredictable swings between severe hyperglycaemia and hypoglycaemia despite intensive therapy. Such erratic profiles amplify exposure to both hypoglycaemia-related autonomic and electrical instability and hyperglycaemia-driven endothelial dysfunction, thereby compounding atherosclerotic progression and risk of sudden cardiac events [41, 42].

### 4.3 Insulin Resistance in T1DM: “Double Diabetes”

Although T1DM is classically defined by absolute insulin deficiency, many patients develop insulin resistance, especially with ageing or weight gain, a phenomenon termed “double diabetes”. This state combines autoimmunity with metabolic syndrome, amplifying risk through visceral adiposity, hypertension, dyslipidaemia, and systemic inflammation [43, 44]. Insulin resistance, often quantified by the estimated glucose disposal rate (eGDR), independently predicts MACE and mortality in T1DM [45].

### 4.4 Obesity

The prevalence of raised body mass index (BMI) in T1DM is increasing in parallel with population trends. Excess adiposity worsens insulin resistance, promotes dyslipidaemia and hypertension, and elevates systemic inflammation [25, 46]. This clustering of cardiometabolic factors attenuates the benefits of intensive insulin therapy and further increases the risk of vascular disease.

### 4.5 Hypertension

In T1DM, especially in those with longer diabetes duration and diabetic kidney disease (DKD), hypertension is common. Elevated blood pressure accelerates endothelial damage and atherosclerosis, increasing risks of stroke, MI, and HF [47]. However, registry data reveal frequent undertreatment and poor attainment of blood pressure targets in youth and young adults with T1DM [48].

### 4.6 Dyslipidaemia

Dyslipidaemia in T1DM often presents as elevated triglycerides, increased small dense LDL, and reduced HDL functionality. Even in patients with reasonable glycaemic control, lipoprotein abnormalities persist, reflecting qualitative differences in lipid metabolism in contrast to patients with T2DM [49, 50]. Elevated cholesterol levels contribute substantially to the atherosclerotic burden in T1DM and account for ~20% of ASCVD events [51].

### 4.7 Smoking

Cigarette smoking markedly increases cardiovascular risk in T1DM by worsening endothelial dysfunction, inflammation, thrombosis, and lipid abnormalities. Its effects are synergistic with hyperglycaemia, further accelerating vascular injury [52]. Smoking cessation remains one of the most impactful interventions in this population.

### 4.8 Family History and Genetics

A family history of premature ASCVD increases CVD risk in T1DM, reflecting both genetic predispositions and shared environmental factors [53, 54]. Genome-wide studies suggest that polymorphisms in inflammatory and vascular genes may modulate risk, but their translation into clinical risk prediction remains limited.

### 4.9 Gender Predominance

Unlike the general population, where premenopausal women enjoy relative cardiovascular protection, women with T1DM experience equal or greater risk than men. This paradox is linked to poorer glycaemic control, higher prevalence of hypertension and dyslipidaemia, and stronger inflammatory activation in women with T1DM [55]. Additionally, hypertensive disorders of pregnancy, including pregnancy-induced hypertension and preeclampsia, are increasingly recognised as sex-specific risk enhancers that further amplify long-term cardiovascular risk in women with T1DM and should prompt closer postpartum risk factor surveillance and control [52].

### 4.10 Physical Inactivity

Physical activity improves glycaemic control, blood pressure, lipid profiles, and cardiorespiratory fitness, lowering CVD risk in T1DM. However, fear of hypoglycaemia and lack of structured support contribute to persistently high rates of physical inactivity in youth and adults with T1DM [56].

### 4.11 Diabetic Kidney Disease (DKD)

DKD is one of the strongest predictors of CVD in T1DM. Albuminuria and declining renal function reflect systemic endothelial dysfunction and are linked to markedly higher risks of MI, stroke, and HF [12, 37]. Significantly, regression of albuminuria is associated with reduced CVD risk, highlighting the kidney–heart interplay.

### 4.12 Cardiac Autoimmunity

Recent evidence suggests that cardiac autoimmunity predicts subclinical myocardial dysfunction and incident cardiovascular events in T1DM [57, 58].

**Fig. 2.**
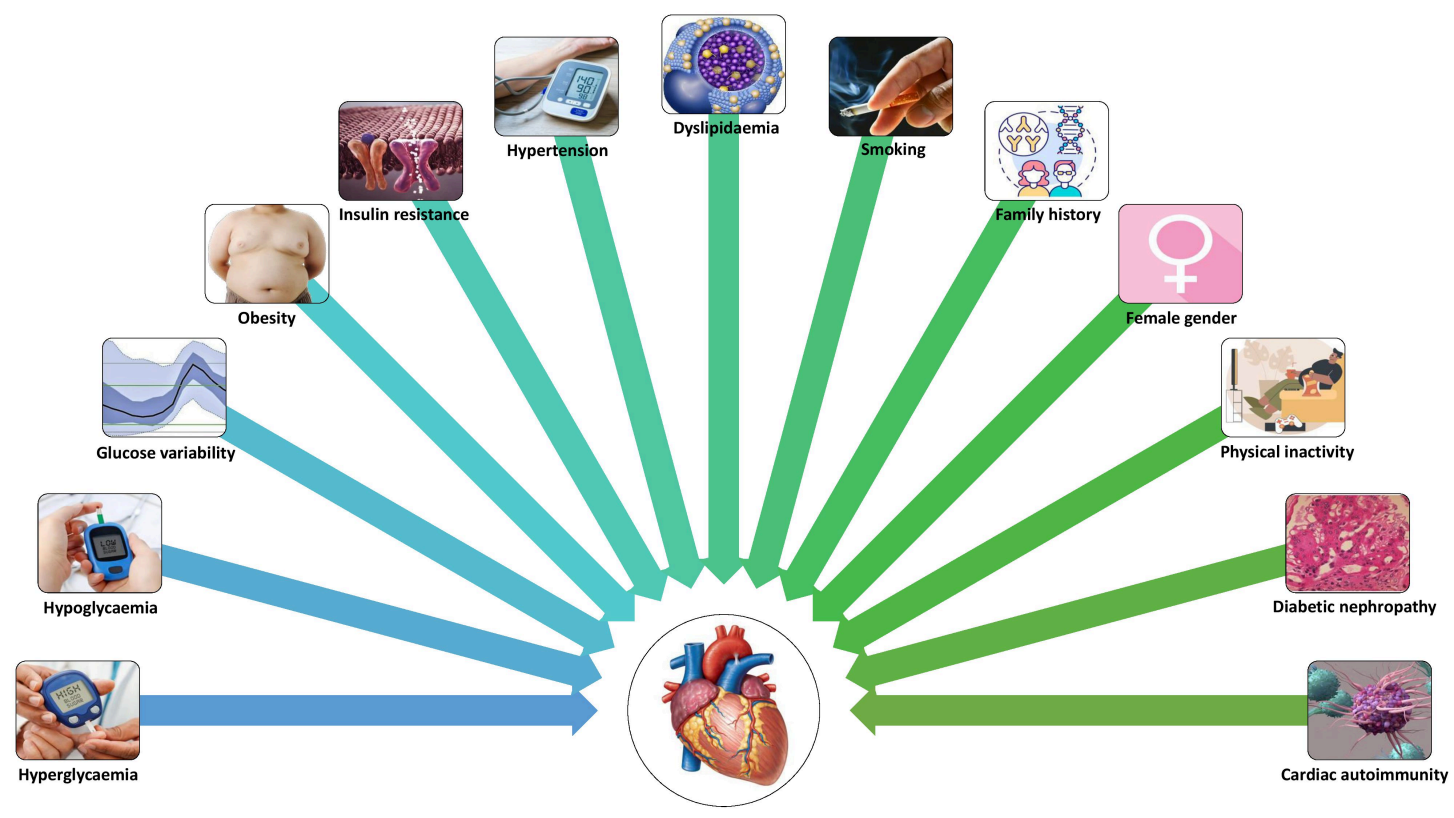
**Risk factors for CVD in T1DM**.

A schematic representation of risk factors for CVD in T1DM is shown in Fig. 2.

## 5. Diagnostic Methods to Detect Subclinical Atherosclerosis in Type 1 Diabetes

### 5.1 Carotid Doppler Ultrasound

Carotid intima–media thickness (cIMT), assessed by carotid Doppler ultrasound, is a widely used non-invasive marker of early atherosclerosis. Multiple studies show that cIMT is increased in children, adolescents, and adults with T1DM compared with controls, correlating with glycaemic control, diabetes duration, and other CVD risk factors [55, 59]. Importantly, cIMT progression predicts future coronary events in long-term follow-up, confirming its prognostic validity [2]. Recent meta-analyses suggest its role in identifying subclinical vascular disease, though variability in methodology limits standardisation [9].

### 5.2 Coronary Artery Calcium Score (CACS)

CACS, performed by non-contrast cardiac CT, quantifies coronary calcification and serves as a predictor of coronary events. Adults with T1DM, particularly those with long disease duration or poor glycaemic control, exhibit higher CACS than non-diabetic peers [60]. However, contemporary data suggest that reliance on calcification alone may underestimate risk in T1DM, where non-calcified plaque burden is substantial [59]. Combining CACS with risk engines such as the Steno T1DM Risk Engine improves event prediction in statin-naïve T1DM populations [61].

### 5.3 Coronary Computed Tomography Angiography (CCTA)

CCTA enables direct visualisation of both calcified and non-calcified coronary plaques. Recent multicentre studies demonstrate that individuals with T1DM have greater plaque volume and higher-risk features, even at younger ages, compared with non-diabetic controls [62]. Importantly, in T1DM, CCTA reveals a high non-calcified plaque burden, supporting its role as a more sensitive modality for risk stratification when CACS is low, but clinical suspicion remains high [44].

### 5.4 Ankle–Brachial Index (ABI)

ABI screening for PAD is a simple and inexpensive procedure. Reduced ABI values are more prevalent in T1DM than in non-diabetic populations, correlating with diabetes duration, reduced metabolic control, and microvascular target organ damage [63]. While not specific for early disease, ABI offers useful prognostic information on systemic atherosclerosis.

### 5.5 Magnetic Resonance Imaging (MRI)

Cardiac and vascular MRI offer high-resolution assessments of arterial wall thickness, plaque composition, and functional measures, including arterial stiffness. Emerging work in T1DM demonstrates MRI’s ability to detect subtle myocardial structural and functional changes before overt disease [64].

### 5.6 Future and Emerging Methods

Recent innovative modalities, like the detection of subclinical CVD in T1DM, have improved outcomes. Advanced ultrasound elastography provides a real-time assessment of arterial stiffness. Additionally, circulating biomarkers, metabolomics, and proteomics are under investigation for early risk stratification. The Cardiovascular Repository for Type 1 Diabetes (CaRe-T1D) consortium is integrating imaging, biomarkers, and genomics to refine cardiovascular risk prediction in T1DM [5]. Table 1 (Ref. [2, 3, 7, 8, 9, 10, 12, 13, 19, 20, 21, 23, 24, 27, 28, 29, 31, 32, 33, 34, 39, 43, 44, 46, 51, 52, 54, 55, 56, 58, 59, 60, 61, 65, 66, 67, 68]) provides a summary of the evidence for various diagnostic methods to detect subclinical atherosclerosis in T1DM.

**Table 1.** **Summary of the diagnostic methods to detect subclinical atherosclerosis in T1DM**.

Diagnostic method	Population & Study	Outcomes assessed	AUC or C-statistic	Sensitivity/Specificity/Calibration	Evidence grade
1. CAROTID INTIMA–MEDIA THICKNESS (cIMT)					
Carotid Doppler Ultrasound (cIMT)	DCCT/EDIC T1DM cohort (n = 1116), 12-year follow-up [58]	Coronary artery disease events, MI, stroke, cardiac death	0.718 (95% CI: 0.650–0.785) [2]	Sensitivity: 71% Specificity: 60% Accuracy: 62.7% [2] Cut-off: 0.95 mm	Level A (Prospective cohort)
cIMT progression	DCCT/EDIC long-term follow-up [67]	Future coronary events, CVD mortality	HR for CVD events increases with cIMT progression	cIMT progression predicts future coronary events (*p * < 0.05)	Level A (Long-term RCT follow-up)
cIMT in T1DM vs controls	Multiple pediatric and adult T1DM studies [9, 51, 52]	Subclinical atherosclerosis, correlation with HbA1c, diabetes duration	0.67–0.79 depending on population [3]	Significantly elevated in T1DM vs controls (*p * < 0.001) [19] cIMT: 0.70 ± 0.11 mm (with complications) vs 0.63 ± 0.09 mm (without) [19]	Level B (Meta-analyses, systematic reviews)
2. CORONARY ARTERY CALCIUM SCORE (CACS)					
CACS by non-contrast cardiac CT	DCCT/EDIC T1DM cohort, 7–9 years post-DCCT, 10–13 year follow-up [20]	MACE, CVD events, mortality	CAC score categories predict events (*p * < 0.0001)	Sensitivity: 80% Specificity: 92% at CAC ≥400 [20] 70% had CAC = 0 (low event rate)	Level A (Prospective cohort)
CACS + ST1RE	Statin-naïve T1DM adults [12, 61]	Cardiovascular risk stratification, event prediction	Improved discrimination when combined with risk engines	CAC >100 associated with subsequent CVD and MACE after adjustment for HbA1c [21]	Level B (Validation studies)
CACS in T2DM (comparative data)	T2DM cohort for reference [7, 10]	CVD mortality, cardiovascular events	AUC varies by population	Sensitivity: 94% (95% CI: 89–96%) Specificity: 34% (95% CI: 24–44%) at CAC ≥10 for mortality/CV events [7]	Level B (Meta-analysis)
3. CORONARY COMPUTED TOMOGRAPHY ANGIOGRAPHY (CCTA)					
CCTA for CAD diagnosis	General diabetic population (mixed T1DM/T2DM) [8]	Obstructive CAD (≥50% stenosis), severe CAD (≥70% stenosis)	AUC: 0.826 (95% CI: 0.717–0.873) for CAD AUC: 0.909 (95% CI: 0.864–0.943) for CCTA-SS [8]	Sensitivity: 87.36% Specificity: 93.48% PPV: 98.15% NPV: 65.15% Accuracy: 88.60% [8]	Level B (Diagnostic accuracy studies)
CCTA plaque detection vs OCT	Consecutive patients with chest pain (n = 40) [23]	Coronary plaque detection (calcified, non-calcified, mixed)	Excellent diagnostic performance	Sensitivity: 92% Specificity: 98% PPV: 84% NPV: 99% Accuracy: 93% [23]	Level B (Section-to-section comparison)
CCTA in T1DM patients	T1DM multicentre studies [13, 54, 56]	Plaque volume, high-risk features, calcified vs non-calcified plaque burden	T1DM shows a higher non-calcified plaque burden vs T2DM [56]	More sensitive than CACS when CACS low but clinical suspicion high [56] Focal and fibrolipidic pattern in T1DM [24]	Level B (Comparative cohort studies)
FACTOR-64 trial	900 diabetic patients (12% T1DM) [21]	MACE incidence, screening utility	CCTA-based screening vs standard care	Risk stratification and therapy adjustment based on findings	Level B (RCT with mixed population)
4. ANKLE–BRACHIAL INDEX (ABI)					
ABI for PAD screening	Diabetes mellitus cohort (n = 99, 194 limbs) [27, 31, 39]	PAD detection	AUC: 0.87–0.89 range [29, 60]	Sensitivity: 35.48% Specificity: 97.55% PPV: 73.33% NPV: 89.83% Accuracy: 87.63% [31, 39] LR+: 14.46 LR-: 0.66 [39]	Level B (Diagnostic accuracy studies)
ABI (general PAD diagnosis)	Structured review, multiple studies (n = 2043) [29, 60]	PAD detection (≥50% stenosis)	–	Sensitivity: 15–79% (variable) Specificity: 83.3–99.0% Accuracy: 72.1–89.2% [29, 60] ABI ≤0.90 cutoff [29]	Level A (Structured review)
ABI vs TBI comparison	Meta-analysis (35 studies for ABI, 9 for TBI) [43]	PAD detection (≥50% stenosis)	ABI: DOR = 16.5 (95% CI: 11.5–23.6) TBI: DOR = 13.1 (95% CI: 7.0–24.8) [43]	ABI: Sens 61%, Spec 92% TBI: Sens 81%, Spec 77% TBI better sensitivity in calcification [43]	Level A (Meta-analysis)
ABI for MAC detection (T1DM)	Type 1 diabetic cohort [28, 33]	Medial arterial calcification on X-ray	–	ABI >1.30: Sensitivity: 14–29.9% Specificity: 97.7–99% PPV: 93% Accuracy: 55–62.2% [28, 33]	Level B (T1DM-specific cohort)
5. PULSE WAVE VELOCITY (PWV)/ARTERIAL STIFFNESS					
Carotid–femoral PWV (cfPWV)	T1DM cohort (FinnDiane study) [32]	Albuminuria progression, eGFR decline ≥30%, CV event, all-cause mortality, composite renal outcome	Significant association with outcomes (*p * < 0.05 for multiple endpoints)	cfPWV higher in T1DM vs healthy controls [32, 44] Gold standard measure of arterial stiffness [32]	Level B (Prospective cohort study)
cfPWV in long-standing T1DM	T1DM patients ≥10 years duration, free from CVD [44]	Association with STENO risk score, CV imaging outcomes, CAC score	Strong association with STENO risk score [44]	Discriminative for premature arterial stiffening Cutoff: 6.9 m/s for PD patients (reference) [39]	Level B (Cross-sectional with comparisons)
PWV in pediatric T1DM	Children and adolescents with T1DM [34, 46]	Early CVD indicators, correlation with glycemic control	–	Increased PWV in youth with <50% time in range as a predictor of CV morbidity and mortality [46]	Level C (Observational pediatric studies)
6. MAGNETIC RESONANCE IMAGING (MRI)					
Cardiac and vascular MRI	Emerging T1DM studies [68]	Arterial wall thickness, plaque composition, arterial stiffness, myocardial changes	–	High-resolution assessment detects subtle myocardial structural/functional changes before overt disease [68]	Level C (Emerging modality)
7. RISK PREDICTION MODELS (for context)					
Steno Type 1 Risk Engine (ST1RE)	T1DM cohorts, external validation [55, 59, 65]	5 to 10-year CVD risk, MACE	C-statistic: 0.71–0.78 (varies by population and validation) [59]	Superior calibration vs general population scores: Good discrimination for CV and microvascular risk [65]	Level A (Validated T1DM-specific model)
Swedish NDR Score	Swedish T1DM registry [55]	5-year CVD risk	Strong calibration, robust external validation [55]	Widely applied in Scandinavian practice	Level A (Registry-based model)
General population scores	T1DM patients [66]	CVD risk estimation	Consistently underestimate risk in T1DM [66]	Not recommended for T1DM [66]	Level A (Consensus)

AUC, area under the curve; C-statistic, Concordance statistic; cIMT, carotid intima–media thickness; DCCT/EDIC, diabetes control and complications trial/epidemiology of diabetes interventions and complications; HR, hazard ratio; CVD, cardiovascular disease; T1DM, type 1 diabetes mellitus; T2DM, type 2 diabetes mellitus; HbA1c, glycosylated haemoglobin; CACS, coronary artery calcium score; CAC, coronary artery calcium; CT, computed tomography; MACE, major adverse cardiovascular events; ST1RE, Steno Type 1 Risk Engine; CCTA, coronary computed tomography angiography; CAD, coronary artery disease; OCT, optical coherence tomography; RCT, randomized controlled trial; ABI, ankle–brachial index; PAD, peripheral artery disease; TBI, toe–brachial index; MAC, medial arterial calcification; cfPWV, carotid–femoral pulse wave velocity; NDR, national diabetes register.

### 5.7 Cardiovascular Risk Prediction in T1DM

Precise prediction of cardiovascular risk in T1DM continues to be a challenge. General population or T2DM scores consistently suggest absolute risk due to the unique profile of early-onset disease, prolonged glycaemic exposure, autoimmune pathogenesis, and distinct vascular mechanisms [65].

Steno Type 1 Risk Engine (ST1RE): ST1RE incorporates ten variables—Age, Sex, Diabetes duration, HbA1c, Systolic Blood Pressure, LDL cholesterol, Estimated Glomerular Filtration Rate, Albuminuria, Smoking status, and Exercise habits. It demonstrates superior calibration and discrimination compared with general or T2DM-based scores [69]. Its utility extends to identifying individuals with both elevated cardiovascular and microvascular risk, thereby guiding the intensification of treatment.

Swedish National Diabetes Register (NDR) Score: The NDR model estimates 5-year CVD risk using routinely collected clinical factors and has undergone robust external validation. It is widely applied in Scandinavian practice and demonstrates strong calibration across diverse populations [66].

Other T1DM-Specific Models: The EURODIAB (EUROpe and DIABetes), Fremantle, and Epidemiology of Diabetes Complications (EDC) models incorporate T1DM-specific variables, including duration and microvascular complications. While showing moderate-to-good discrimination, they require recalibration in multi-ethnic and contemporary populations [67]. The Scottish Care Information (SCI) Diabetes model, derived from registry data, incorporates socioeconomic status and demonstrates strong predictive performance at 5 and 10 years [11].

General Population Scores: Equations such as ASCVD, the United Kingdom Prospective Diabetes Study (UKPDS), Framingham, QRISK3, and pooled joint-society scores consistently underestimate risk in T1DM and are not recommended for clinical use in this population [70]. European Society of Cardiology (ESC) 2019 guidelines instead classify most adults with longstanding T1DM or complications as high or very high risk, though this framework is based on consensus rather than formal validation [6].

Contemporary cohorts show underuse of statins, delayed initiation of antihypertensives, and suboptimal attainment of treatment targets in young adults with T1DM, even when risk models or imaging indicate elevated long-term risk [14].

## 6. Prevention of Cardiovascular Disease in T1DM

### 6.1 Lifestyle Interventions

Lifestyle measures remain foundational. Mediterranean-style diets, rich in fibre and low in saturated fats, support both metabolic and vascular health. Structured aerobic and resistance exercise improves insulin sensitivity, blood pressure, adiposity, and lipid profiles, while smoking cessation is essential given the synergistic risk amplification between tobacco and diabetes [12]. Behavioural support improves long-term adherence.

### 6.2 Pharmacologic Interventions

Adequate and appropriate insulin therapy is key to achieving optimal glycemia, with DCCT/EDIC reaffirming the “legacy effect” of HbA1c in reducing long-term CVD risk [71]. Statins are recommended for most adults over 40 and for younger individuals with additional risk factors, significantly reducing ASCVD events. Antihypertensives like ACE inhibitors or ARBs offer renal and cardiovascular protection, especially in the presence of albuminuria. Low-dose aspirin is reserved for secondary prevention due to the risk of bleeding [72].

### 6.3 Emerging Therapies

Continuous glucose monitoring (CGM) and hybrid closed-loop insulin delivery systems reduce glycaemic variability and hypoglycaemia, both of which are linked to vascular outcomes [73]. Novel pharmacotherapies, such as SGLT2 inhibitors and GLP-1 receptor agonists, show cardiovascular benefits in T2DM but remain investigational in T1DM due to safety concerns, including euglycemic ketoacidosis [73]. Adjunctive therapies under study include verapamil for β-cell preservation and immunotherapies targeting autoimmune processes [73].

### 6.4 Mechanistic and Non-Traditional Risk Factors

Severe hypoglycaemia is an independent predictor of cardiovascular events [5]. Remnant cholesterol accounts for a notable proportion of ASCVD risk in diabetes [70]. MASLD is increasingly prevalent in T1DM and independently raises CVD risk [67]. Distinct lipoprotein biology, including HDL dysfunction and *ApoB*–HDL imbalance, contributes to residual risk [70]. Women with T1DM experience greater relative excess cardiovascular risk than men, losing the protective female advantage observed in non-diabetic populations [6]. Adult-onset T1DM also carries high cardiovascular and mortality risk, suggesting the need for vigilance across the age spectrum [14].

### 6.5 Imaging and Biomarker-Guided Stratification

Innovative imaging techniques complement risk stratification scores. Coronary CT imaging enables the early detection of plaque burden, while cIMT and measures of arterial stiffness serve as practical indicators of subclinical CVD [50]. Advanced MRI techniques can uncover initial signs of myocardial remodelling. When combined with biomarkers—such as inflammatory markers, indices of endothelial dysfunction, and novel metabolomic profiles—these imaging modalities offer significant potential for precise risk stratification [51].

### 6.6 Health Economics of CVD Reduction in T1DM

Premature CVD in T1DM imposes substantial economic costs from hospitalisations, disability, and lost productivity. Health economic evaluations demonstrate that early management of glycemia, blood pressure, and lipid levels can significantly reduce long-term healthcare costs while improving quality-adjusted life years [50]. The routine use of validated risk prediction models, statins, antihypertensives, and smoking cessation initiatives further improves cost-effectiveness [59]. Nonetheless, ongoing undertreatment—especially among women and younger individuals—continues to undermine the full economic and clinical potential of these strategies [6].

**Fig. 3.**
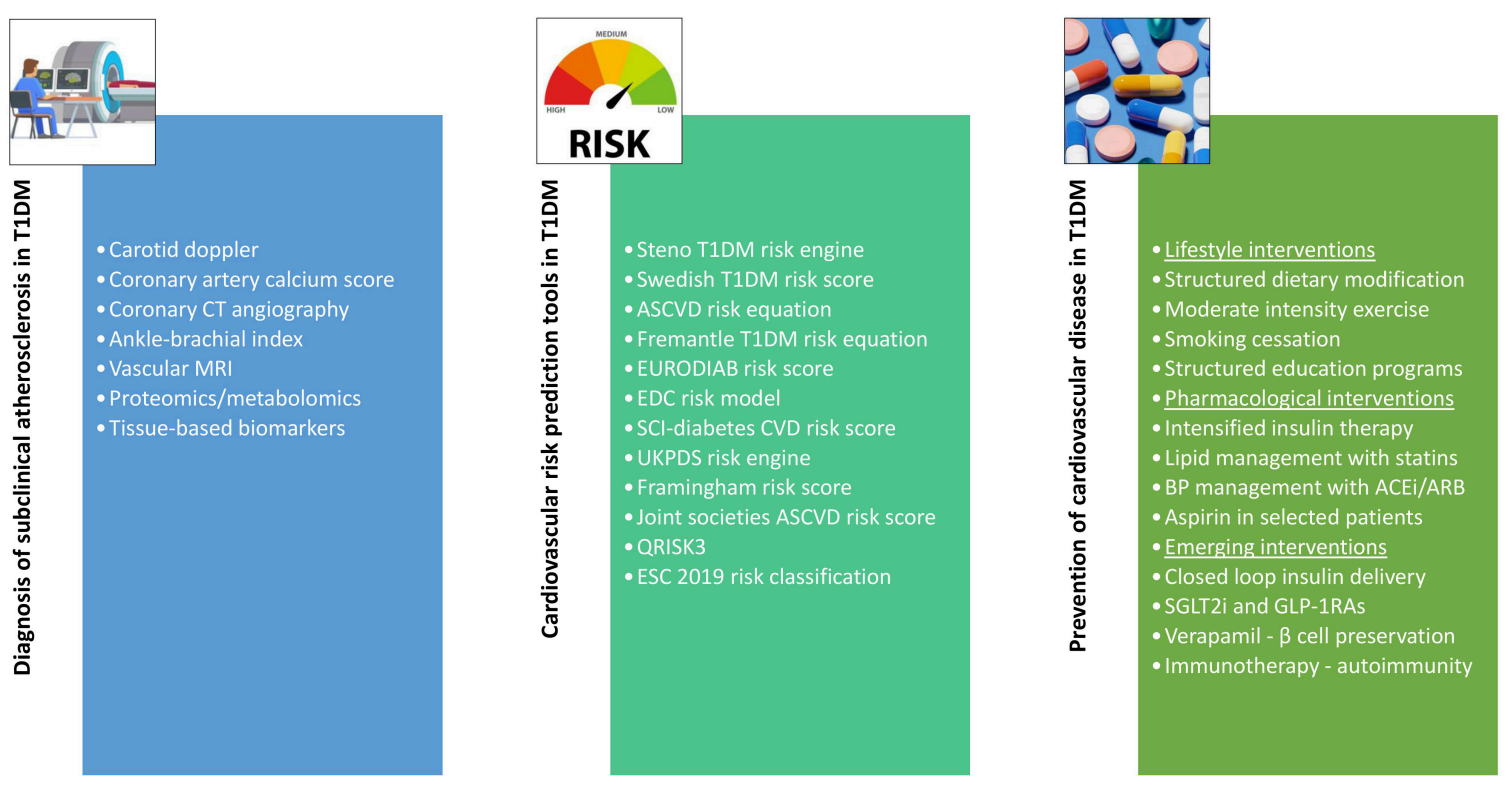
**Algorithm for diagnosis, risk prediction, and prevention of CVD among patients with T1DM**. CT, computed tomography; MRI, magnetic resonance imaging; T1DM, type 1 diabetes mellitus; ASCVD, atherosclerotic cardiovascular disease; EURODIAB, EUROpe and DIABetes; EDC, Epidemiology of diabetes complications; SCI, Scottish Care Information; UKPDS, the United Kingdom Prospective Diabetes Study; ESC, European Society of Cardiology; BP, blood pressure; ACEi, angiotensin-converting enzyme inhibitor; ARB, angiotensin II receptor blocker; SGLT2i, sodium-glucose co-transporter 2 inhibitor; GLP-1RA, glucagon-like peptide-1 receptor agonist.

An algorithm for diagnosis, risk prediction, and prevention of CVD among patients with T1DM is elaborated in Fig. 3.

## 7. Conclusions

Cardiovascular disease is a leading cause of morbidity and mortality in T1DM, emerging earlier and with greater severity than in the general population. Risk is magnified by chronic hyperglycaemia, insulin resistance, hypertension, dyslipidemia, MASLD, hypoglycaemia, and gender specific factors. Women and those diagnosed in childhood face disproportionate lifetime risk. However, due to a lack of T1DM–specific data, the scope is limited and warrants further research. Despite advances in technology, imaging, and pharmacology, residual cardiovascular burden persists. Precision medicine strategies that incorporate T1DM-specific risk models, newer imaging techniques, biomarkers, and gender-specific strategies reduce health disparities. Early, lifelong intervention remains the cornerstone of improving both cardiovascular outcomes and long-term economic sustainability.
